# Dr. Hunter’s Claims Vindicated by Himself

**Published:** 1852-07

**Authors:** 


### Editorial.

### DR. HUNTER'S CLAIMS VINDICATED BY HIMSELF.

We have never intended taking it upon ourself to settle the claims of Drs. Hunter and Allen; and, as a journalist, we have only given notice of such improvements as have been shown us from time to time, and we have tried to do this faithfully to the profession. Dr. Allen, we knew, had been experimenting for some time to get a cement which would unite the teeth and plate. On more than one occasion had he exhibited to us specimens of his work, which although sufficient to excite hopes of ultimate success, yet were not perfect enough to justify a special notice to the profession. When at last his work showed evidence of utility, we made known the fact, but we had no thoughts of thereby injuring the professional reputation of any one, nor of taking one iota of credit from any member of the profession. As to originality of conception in the uniting of teeth into a continuous block, we presume that neither Dr. Allen or Hunter, could claim priority. But as to successful and satisfactory results both could claim a good deal. But it appears that we had great "exaltation" in the thought that we had got an improvement which would "entirely supercede what are called block-teeth. It appears to have struck Dr. Hunter as something very strange, that we could exult over this, and yet not be the discoverer; but so it was, in the good of the profession we forgot our selfishness. But the following quotation from the Doctor's "vindication by himself," gives us the first inkling of the cause of his discontent. In our notice of Dr. Allen's improvement, the Dr. remarks, that "he professes never to have heard of my improvement which was designed and perfected to overcome the difficulties of block work."

This, although true, gives but a one-sided view of the case. We had heard through Dr. King, that Dr. Hunter had made an improvement in his block work, and that the improvement consisted in uniting single teeth by means of a cement or body, and that these blocks were soldered after thus made to the plate. None of the particulars were given, and the information thus received was obtained in the following manner. We were describing in our office to our brother and Dr. King, a specimen which Dr. Allen had exhibited us of his work, where the teeth were united to each other and the plate, and expressed an opinion of its practicability, and at this time Dr. King remarked, that Dr. Hunter had made an improvement in block work, which was the first we heard of his improvement, but said that he was not at liberty to say anything about it—that it was still a secret with Dr. Hunter, &c. Under this impression of secrecy, we never felt at liberty to speak of it editorially, until the last issue of the Register, and this privilege was only granted by Dr. Hunter himself, a short time before the last No. of the Register went to press, and even then on condition that we should describe the specimen sent to the World's Fair.

There has been no time in which we should not as freely have spoken of Dr. Hunter's claims as Dr. Allen's, or any other member of the profession. As Editor of the Register, we feel it our duty to give the profession every thing which is new and of service. And however gratifying it might be to have made these improvements ourself, yet we are glad to see them anyhow, and glad to have them in our city, even should they be by our competitors in business. We feel that so far as Dr. Hunter has cause of complaint, he can more blame himself than us. The profession who have noticed the run of affairs, know that the West has not been considered good ground on which to settle claims so important to the profession; either this, or that, the Register would not suit to fight so great a battle in.

In relation to Steemer's testimony, our assertion was made in connection with the fact that he was an interested witness and as such his testimony could not be taken. We say this in justice to an individual of whom we know nothing save what this affair has developed. We suppose if Mr. Steemer, and Drs. Hunter and Allen, were to attempt befoie a court of justice to prove priority, the testimony of neither would be allowed. We do not rely on Dr. Allen's assertion any more than on Dr. Hunter's. We had seen enough to satisfy us that Dr. Allen deserved credit for the perfection to which he had brought his experiments. We had not seen Dr. Hunter's. In conclusion we would say, that we have only noticed this expose of Dr. Hunter, thus far, to correct the false impression which his article is calculated to make, particularly in relation to ourself. Had we room in the pages of the Register, we should publish his article and set the Dr. right in relation to the Faculty of the Ohio College of Dental Surgery. The profession, however, who have read the card from the Faculty and students, can easily see that they have done Dr. Hunter no injustice, but only aimed to correct the impression which the Dr. had made, that Dr. Allen's improvement was worthless. We cannot think it necessary in Dr. Hunter proving priority of improvement, to detract from the merits of Dr. Allen's. If worthless, no prior claim need be established.

Since we commenced this article, Dr. Hunter showed us a specimen of his work done on gold plate alloyed with platinum, which we can with propriety say, looks remarkably well. It is a duplicate of one made for the mouth, and we are assured the body and gum is flowed on at one heating, and that no contraction takes place in the operation. We would here give not a "prophetic doubt," but a *prophetic hope*, that these improvements will still be *perfected* more and more until no one shall have a *doubt* of their utility.

We would here, also, correct an error in relation to Dr. Allen's improvement which we find many labor under,—which is, that it requires some half dozen heatings to finish an operation. After soldering the teeth to the plate as we usually do, it only requires two or at the most three.

We hope, before long, to present the profession with a method of dispensing with a separate gum color. This Dr. Hunter appears already to have obtained; this will diminish the number of heatings. We hope that Dr. Hunter's "*disinterestedness*" will induce him to let the profession have the benefit of some of his secrets. If he would only get them *patented*, we could at least learn what they are.

